# Crosstalk Between Inflammasome Signalling and Epithelial-Mesenchymal Transition in Cancer and Benign Disease: Mechanistic Insights, Context-Dependence, and Therapeutic Opportunities

**DOI:** 10.3390/cells14201594

**Published:** 2025-10-14

**Authors:** Abdul L. Shakerdi, Emma Finnegan, Yin-Yin Sheng, Karlo Vidovic, Jessica M. Logan, Mark P. Ward, Sharon A. O’Toole, Cara Martin, Stavros Selemidis, Doug Brooks, John J. O’Leary, Prerna Tewari

**Affiliations:** 1 Discipline of Histopathology, School of Medicine, Trinity Translational Medicine Institute (TTMI), Trinity College Dublin, D02 PN40 Dublin, Ireland; shakerda@tcd.ie (A.L.S.); finnege3@tcd.ie (E.F.); shengyi@tcd.ie (Y.-Y.S.); vidovick@tcd.ie (K.V.); wardm6@tcd.ie (M.P.W.); shotoole@tcd.ie (S.A.O.); cmartin3@tcd.ie (C.M.); olearyjj@tcd.ie (J.J.O.); 2 Clinical and Health Sciences, University of South Australia, Adelaide, SA 5001, Australia; jessica.logan@unisa.edu.au (J.M.L.); doug.brooks@unisa.edu.au (D.B.); 3 School of Health and Biomedical Sciences, RMIT University, Melbourne, VIC 3083, Australia; stavros.selemidis@rmit.edu.au; 4 Eurofins Clinical Genetics Ireland, D08 NHY1 Dublin, Ireland

**Keywords:** inflammasome, epithelial–mesenchymal transition, metastasis, fibrosis, therapeutic targeting

## Abstract

Epithelial-mesenchymal transition (EMT) and inflammasome signalling are intercon-nected processes which underpin tumour progression, metastasis, and therapeutic re-sistance. Inflammasomes such as NLRP3 encourage pro-inflammatory states (IL-1β, IL-18, NF-κB) and the activation of signalling pathways like TGF-β that promote mes-enchymal traits crucial for EMT. EMT transcriptional programmes can then in turn modulate the inflammasome via NF-κB/TGF-β signalling, creating self-perpetuating mechanisms of cellular plasticity and dysregulated therapeutic response. We have re-viewed the mechanistic evidence for EMT–inflammasome crosstalk in cancer and discussed the potential therapeutic implications. The function of the EMT-inflammasome axis is clearly context-dependent, with the cancer type, stage, and the complexity of the tumour microenvironment heavily contributing. The crosstalk between EMT and the inflammasome is an overlooked mechanism of tumour evolution, and targeting inflammasomes like NLRP3, or their downstream signalling pathways, offers a promising therapeutic avenue, with the objective of inhibiting metastasis and overcoming drug resistance.

## 1. Introduction: Two Molecular Processes Controlling Tumour Cell Plasticity

Cancer progression is dependent on more than simply the accumulation of genomic lesions. Indeed, it requires cellular plasticity and phenotypic changes that allows malignant cells to invade, evade, and adapt to conditions at their primary and distant sites. The epithelial–mesenchymal transition (EMT) and inflammasome signalling have been identified as two key components of tumour cell versatility, yet are often studied as separate pathological processes. EMT is a dynamic cellular programme by which epithelial cells lose their polarity and cell-cell adhesion, yielding a more mesenchymal profile that enables motility and tissue infiltration [1]. This phenotypic plasticity is dependent on a complex network of transcriptional regulators, epigenetic modifiers, and extracellular cues within the tumour microenvironment [2,3,4]. In physiological states, EMT is involved in processes such as embryogenic gastrulation, wound healing, and tissue remodelling [5]. Conversely, EMT is exploited by cancer cells during the metastatic cascade, as it confers invasive and migratory potential upon epithelial cells [6]. EMT is also implicated in the pathogenesis of various non-malignant diseases, such as chronic fibrotic disorders [7], inflammatory rheumatic disorders [8], age-related macular degeneration [9], and reproductive disorders such as adenomyosis and endometriosis [10,11]. Emerging evidence suggests that components of the innate immune system, including the inflammasome complex, play a significant regulatory role in EMT [12]. Inflammasomes are cytosolic multimeric complexes that respond to pathogenic or stress signals through the activation of caspases and the maturation of pro-inflammatory cytokines [13]. The inflammasome plays a crucial physiological role in innate immunity by activating inflammatory responses to pathogens [14], yet its dysregulation contributes to the development of various diseases such as cancer, gout, atherosclerosis, and neurodegeneration [15]. This review aims to critically examine the emerging interplay between inflammasome signalling and EMT in malignant and non-malignant diseases and consider its potential as a therapeutic target.

### 1.1. Epithelial-Mesenchymal Transition: A Complex Molecular and Transcriptional Programme

The changes to cellular identity seen in EMT ensue as a result of molecular changes and the activity of various transcriptional regulators. Molecular changes identified include the repression of epithelial markers such as E-cadherin, occludin, and specific cytokeratins, with concomitant upregulation of mesenchymal genes including N-cadherin, vimentin, fibronectin, and matrix metalloproteinases (MMPs) [16]. These changes are facilitated by EMT transcription factors such as Snail family transcriptional repressor 1 (SNAIL) and Snail family transcriptional repressor 2 (SLUG) [4,17,18]. Notch signalling is also involved in EMT, mainly due to its role in upregulating transcriptional repressors like SNAIL and SLUG [19,20].

These transcriptional events are accompanied by histological changes. Cells undergoing EMT demonstrate a transition from a cobblestone-like epithelial morphology to an elongated, spindle-shaped mesenchymal phenotype with front-rear polarity, extensive actin cytoskeletal reorganisation, and loss of basement membrane association [16,21]. These morphological changes are due to hemidesmosome loss and switching from integrin β4 to integrins α5β1 and αvβ6 [22]. It is important to acknowledge that EMT is not a binary switch, but instead a spectrum with transitional states that yield hybrid epithelial/mesenchymal phenotypes [23]. EMT has also been linked to the acquisition of cancer stem cell (CSC)-like properties [24]. CSCs are a group of cells characterised by enhanced tumourigenicity, plasticity, and therapeutic resistance, and they are considered the apex of the cellular hierarchy of the tumour [25].

**Figure 1:**
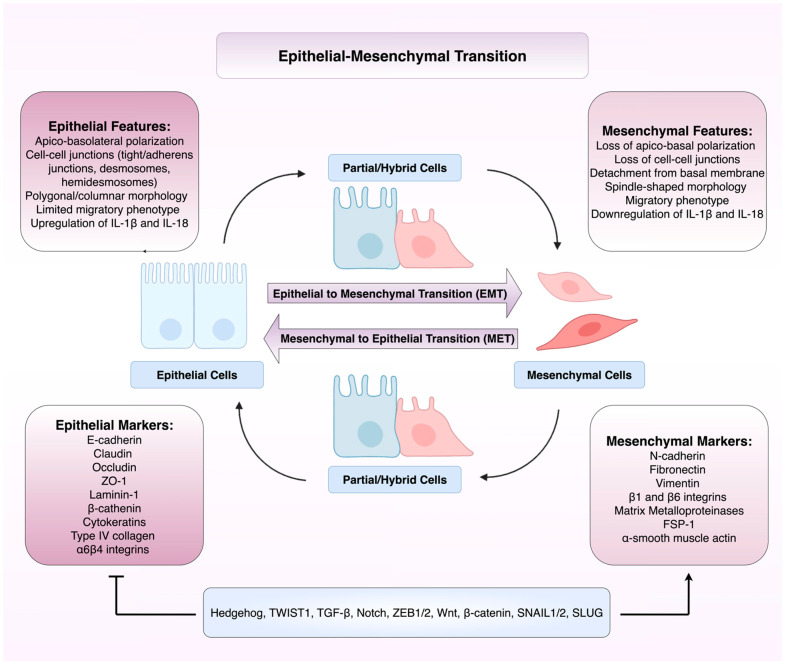
Overview of the phenotypic, molecular, and transcriptional changes in epithelial–mesenchymal transition (EMT) and mesenchymal–epithelial transition (MET). This diagram depicts the bidirectional and dynamic nature of EMT, in which epithelial cells with apio-basolateral polarity and strong intercellular junctions transition through partial/hybrid phenotypes to acquire mesenchymal traits such as front-rear polarity, basement membrane detachment, and increased migratory capacity. Conversely, mesenchymal–epithelial transition (MET) allows mesenchymal cells to regain epithelial morphology and polarity. Figure created using BioRender (version 2025, BioRender.com, Toronto, ON, Canada) and Microsoft PowerPoint (Microsoft Corporation, version 365, Redmond, WA, USA).

EMT is traditionally divided into three distinct subtypes which perform specific biological functions. Type 1 EMT creates cell movement that is not associated with fibrosis or an invasive phenotype, and has roles in embryo formation, implantation, and organogenesis [26,27]. Type 2 EMT is associated with repair events such as wound healing, tissue regeneration and organ fibrosis. This EMT subgroup is associated with inflammation and can lead to tissue fibrosis and organ destruction [26,27]. Type 3 EMT occurs in malignant cells that have undergone genetic and epigenetic changes to promote growth of tumours at their primary site [26,27] with further genetic changes and environmental factors promoting invasion and metastasis [27]. Figure 1 illustrates the dynamic and reversible process of EMT and mesenchymal–epithelial transition (MET), highlighting the shift between epithelial, partial/hybrid, and mesenchymal cell states, characterised by distinct morphological features, marker expression, and regulatory pathways.

### 1.2. Inflammasomes in Cancer

Inflammasomes are multimeric protein complexes that play a crucial role in innate immunity. These molecules assemble in the cytosol after sensing pathogen-associated molecular patterns (PAMPs) or damage-associated molecular patterns (DAMPs) [28]. Three critical components make up canonical inflammasomes, an activated pattern recognition receptor (PRR), apoptosis-associated speck-like protein containing a caspase recruitment domain (ASC) and procaspase-1 [15]. PRRs can be classified as cytosolic or transmembrane receptors, serving as sensor proteins [29]. Transmembrane PRRs can recognise PAMPs and DAMPs, triggering a signalling cascade which recruits cytosolic PRRs, although the cytosolic counterpart can also be activated by intracellular pathogens alone. Multiple PRRs can form inflammasome complexes, including NOD-like receptor family pyrin domain-containing 1 (NLRP1), NLRP3, NOD-like receptor family, caspase activation and recruitment domain–containing 4 (NLRC4) and absent in melanoma 2 (AIM2) [14]. Among the various inflammasomes identified, NLRP3 and AIM2 have emerged among the most extensively characterised in cancer biology. These complexes not only mediate key inflammatory responses but also appear to display distinct, context-dependent roles in regulating tumour progression and EMT. Therefore, this review will primarily focus on NLRP3 and AIM2 inflammasomes in the context of cancer-related EMT and its therapeutic modulation.

Activation of the NLRP3 inflammasome begins with a priming step, where a cytokine receptor or Toll-like receptor (TLR) signalling induces the activation and nuclear translocation of nuclear factor kappa-light-chain-enhancer of activated B cells (NF-kB), which in turn initiates the transcription and translation of NLRP3 and pro-IL1β. Other intracytosolic signals, including lysosomal or mitochondrial disruption, cause NLRP3 to undergo a conformational change via post-translational modifications which allows it to associate with ASC through pyrin domain (PYD) or caspase recruitment domain (CARD) interactions [30,31]. ASC can then recruit caspase-1 through CARD-CARD interactions [32]. Active caspase-1 is a cystine-dependent protease which can cleave precursor cytokines pro-interleukin-1β (pro-IL-1β) and pro-IL-18 to their active states, generating biologically active cytokines [33]. Active caspase-1 can also contribute to an inflammatory form of cell death formally titled pyroptosis [34].

**Figure 2:**
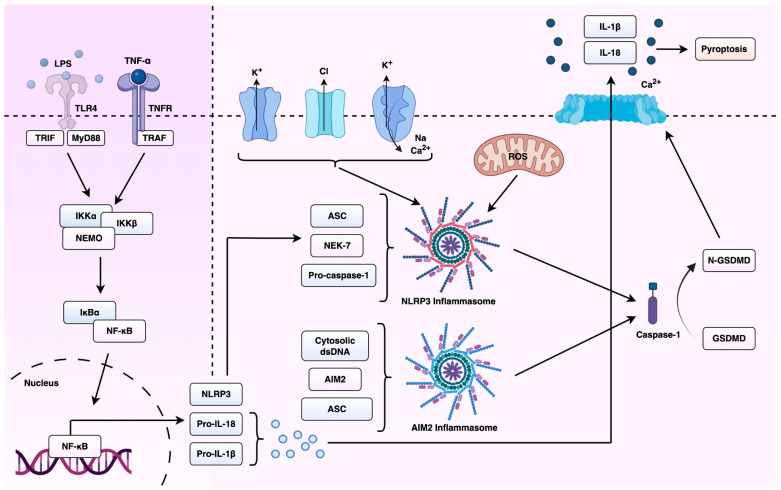
Overview of NLRP3 an AIM2 inflammasome activation and downstream signalling in cancer. This schematic illustrates the canonical activation pathway of the NLRP3 inflammasome. Upon sensing cellular stressors such as ROS, DNA damage, oncogenic mutations, the NLRP3 inflammasome assembles and activates caspase-1. This leads to the cleavage and maturation of pro-inflammatory cytokines IL-1β and IL-18, which shape the inflammatory milieu of carcinomas. Figure created using BioRender (version 2025, BioRender.com, Toronto, ON, Canada) and Microsoft PowerPoint (Microsoft Corporation, version 365, Redmond, WA, USA).

The NLRP3 inflammasome is activated in response to a wide range of stimuli, including nucleic acids, monosodium urate crystals, potassium and chloride flux, and β-glucans [35,36,37,38]. It represents the best studied inflammasome-nucleating sensor and is associated with a variety of inflammatory and autoimmune disorders, including gout, type 2 diabetes [39,40], Alzheimer’s disease [41] and atherosclerosis [42]. Chronic activation of the NLRP3 inflammasome can result in increased production of pro-inflammatory cytokines (IL-1β and IL-18), causing tissue damage and exacerbation of disease symptoms [43]. The understanding of the molecular mechanisms that control NLRP3 activation has become a major research focus, including the therapeutic potential of this complex. A summary of the priming and activation mechanisms of the NLRP3 inflammasome, and its downstream effects on cytokine maturation and pyroptosis, are shown in Figure 2. The targeting of NLRP3 or its downstream signalling pathways offers promise for the development of novel anti-inflammatory drugs, mitigating the progression of inflammasome-associated diseases and improving patient outcomes.

## 2. Molecular Mechanisms Linking Inflammasome Signalling to Epithelial-Mesenchymal Transition in Cancer

There exists evidence implicating the inflammasome signalling in modulating the EMT process both intracellularly and through intercellular communication across several disease states. Early research demonstrated that Il-1β, which is released extracellularly following inflammasome activation, enhances transforming growth factor beta (TGF-β) activity and in turn promotes EMT in human bronchial epithelial cells [44]. IL-1β has also been shown to promote changes associated with EMT, such as E-cadherin downregulation and Snail expression in both gastric and oral cancer cells [45,46]. Angiotensin II is a peptide hormone produced through the action of angiotensin-converting enzyme on angiotensin I [47]. It has been shown to drive hepatocyte EMT by inducing nicotinamide adenine dinucleotide phosphate oxidase 4 (NOX4)-dependent H_2_O_2_ production, resulting in activation of the NLRP3 inflammasome and IL-1β release [48]. IL-1β engages mothers against decapentaplegic homolog (Smad) signalling pathways, orchestrating the epithelial-to-mesenchymal shift. Critically, this work showed that NLRP3 and caspase-1 are required for angiotensin II-induced EMT. The peptide Ang-(1–7) was later discovered to act as a counterregulatory protein that attenuated this process through NLRP3 suppression. Further work illustrated that inflammasome-independent NLRP3 signalling can augment TGF-β signalling intracellularly in renal epithelium. This demonstrated that EMT can be triggered independently of inflammasome oligomerisation, with only the presence of the NLRP3 protein [49]. Several lines of evidence link inflammasome–EMT crosstalk to the pathogenesis of various malignancies and benign diseases, such as fibrotic disorders, diabetic nephropathy, and endometriosis. Thus, the mechanistic crosstalk between inflammasomes and EMT is best viewed as a dynamic feedback circuit: inflammasomes activate EMT through cytokines and signalling networks, while EMT factors remodel inflammasome activity to sustain inflammatory plasticity. This symbiosis produces self-reinforcing loops that underpin lethal properties of cancer including invasion, metastasis, and therapy resistance. We will examine site-specific evidence by which this interplay occurs both intracellularly and intercellularly.

### 2.1. Inflammasome-Driven Modulation of EMT: Tumour-Specific Intercellular Mechanisms

#### 2.1.1. Lung Cancer

Inflammasome pathways modulate EMT in different tissues, but the consequences are highly context-dependent. In lung cancer, chronic carcinogen exposure fosters a persistently inflamed tumour microenvironment which is partially driven by inflammasome action. Specifically, inflammasomes release IL-1β and IL-18, activating NF-κB and hypoxia-inducible factor 1-alpha (HIF-1α) signalling in the tumour microenvironment [50]. The literature recognises HIF-1a as a major regulator of transcription factors which are associated with a mesenchymal prototype, including TWIST, SLUG, SNAIL, Zinc finger E-box-binding homeobox 1 (ZEB1) and Smad-interacting protein 1 (SIP1) [51]. There is also a known correlation between IL-1β and the induction of EMT in lung cancer [52]. Tumour-derived small extracellular vesicles (TEVs) from cells treated with IL-1β have been shown to activate a5β1 signalling, resulting in the upregulation of vimentin and SMAD3, cell migration and the EMT [53]. Additionally, exposure to IL-1β decreases phosphatase and tensin homolog (PTEN) expression, subsequently activating phosphatidylinositol 3-kinase/protein kinase B (PI3K/AKT) signalling and inducing EMT in lung cancer [54].

The tumour microenvironment is deeply involved in the crosstalk between EMT and inflammasome signalling in lung cancer. For instance, tumour-secreted TGF-β shifts neutrophil differentiation from a tumour-suppressive N1 phenotype into a tumour-promoting N2 phenotype [55]. It has been previously demonstrated that neutrophil extracellular traps (NETs) produced in the microenvironment promote NSCLC cell invasion and migration by inducing EMT. NETs are thought to induce the NLRP3 signalling pathway and the metastatic process by downregulating the expression of a specific tumour-suppressing long-coding RNA, MIR503HG [56]. Additional studies have supported these findings, as pre-treatment with NET inhibitors (anti-NE antibody and DNase) effectively reduces the migration and invasion of lung cancer cells [57].

#### 2.1.2. Breast Cancer: NLRP3 as a Promoter of EMT and Therapy Resistance

Studies have shown that NLRP3 and EMT may play an important role for therapeutic resistance in breast cancer. NLRP3 is aberrantly overexpressed in invasive breast ductal carcinoma, particularly in the claudin-low subtype of triple-negative breast cancer (TNBC) [58]. This subtype is known for mesenchymal traits, immune evasion, and poor clinical outcomes [59,60,61]. High NLRP3 expression correlated with worse patient survival, highlighting potential prognostic relevance in breast cancer [58]. In this context, NLRP3 was found to drive EMT and metastatic progression through autocrine IL-1β signalling via a caspase-1-dependent manner. Knockdown of NLRP3 in claudin-low MDA-MB-231 cells suppressed migration, invasion, and EMT. Conversely, overexpression of NLRP3 in MCF-7 cells induced EMT-like changes and invasive behaviour. Rescue experiments supported a causal relationship, as treatment with IL-1β restored EMT in NLRP3-silenced cells. Similarly, IL-1β antibody treatment reversed EMT in NLRP3-overexpressing cells [58]. In vivo, NLRP3 knockdown reduced metastasis in both zebrafish and nude mouse models, whereas its overexpression promoted the formation of metastatic foci in the lungs of mice [58].

One study [62] demonstrated that NLRP3 also contributes to chemoresistance in TNBC by modulating epithelial plasticity and inflammatory signalling. Using gemcitabine-resistant TNBC cell lines, they found that NLRP3 expression was significantly elevated compared to parental lines. Agonism of NLRP3 further reduced sensitivity to gemcitabine. Conversely, inhibition of NLRP3 using the selective antagonist CY-09 restored drug sensitivity and reduced cell viability. NLRP3 upregulation was found to induce a mesenchymal phenotype and to enhance the secretion of IL-1β and activation of Wnt/β-catenin signalling [62]. This was supported by increased phosphorylation of glycogen synthase kinase-3 beta (GSK-3β) at Ser9, indicative of decreased GSK-3β activity, as well as accumulation of β-catenin. Inhibition of Wnt/β-catenin signalling downregulated NLRP3 expression, suppressed IL-1β production, reversed EMT marker expression, and impaired cell survival. This indicates the presence of a regulatory pathway that enables drug resistance through NLRP3-IL-1β-Wnt/β-catenin signalling [62]. These findings reveal that inflammasome activity confers EMT properties and chemoresistance in TNBC, yet also highlight NLRP3 as a potential target to enhance therapeutic efficacy.

#### 2.1.3. Colorectal Cancer: Dualistic and Context-Dependent Roles

Recent investigations into the interplay between EMT and inflammasome signalling in colorectal cancer (CRC) have shown contrasting results, which highlight the nuanced role of the inflammasome in tumour progression. One study [63] examined tissue samples from 43 CRC patients and demonstrated that the activation of the NLRP3 inflammasome correlated with EMT induction and tumour grade. In addition, the expression levels of NLRP3, caspase-1, IL-1β, and NF-κB were markedly elevated in CRC, particularly in high-grade tumours. EMT-associated proteins such as N-cadherin, vimentin, and MMP-9 were upregulated, whereas E-cadherin was negatively correlated with NLRP3 protein levels [63]. In contrast, a mechanistic study using murine in situ models and co-culture systems provided opposing findings. Here, the inhibition of NLRP3 inflammasome activity by transmembrane protein 176B (TMEM176B) facilitated EMT while knockdown of TMEM176B in CT26 murine CRC cells increased the expression of NLRP3 inflammasome proteins and epithelial markers, while reducing the expression of mesenchymal markers [64]. The partial reversal of these effects was observed upon the concurrent knockdown of NLRP3 [64]. This work suggests that in certain contexts, inflammasome activation may instead exert an anti-metastatic effect by reinforcing epithelial integrity.

### 2.2. Intracellular Insights Across Diverse Malignancies

#### 2.2.1. Lung Cancer

One study [65] demonstrated that activation of the NLRP3 inflammasome in A549 lung adenocarcinoma cells, achieved via lipopolysaccharide (LPS) priming followed by adenosine triphosphate (ATP) stimulation, led to a significant upregulation of SNAIL and a downregulation of E-cadherin. This effect was dependent on NLRP3 expression, and indeed, neutralisation of NLRP3 via shRNA reversed these EMT-associated transcriptional and molecular changes. Pharmacological inhibition of downstream inflammasome signalling through caspase-1 inhibition, IL-1 receptor antagonism, and the use of IL-18 binding protein (IL-18BP) produced similar cellular consequences [65]. Additionally, inhibition of the AIM2 inflammasome (a DNA-sensing inflammasome) via luteolin, a natural flavonoid, reduced mesenchymal markers like vimentin and MMP-9 while increasing E-cadherin. These findings indicate that both NLRP3 and AIM2 inflammasome signalling actively induce the EMT programme in lung cancer [66].

#### 2.2.2. Pancreatic Cancer: Regulation of Inflammasome–Emt Axis by lncRNA

Long non-coding RNA (lncRNA) has interestingly been demonstrated to play a role in the regulation of the inflammasome–EMT axis in pancreatic cancer. These represent are transcripts longer than 200 nucleotides that lack protein-coding potential, but play critical roles in regulating chromatin architecture, transcriptional activity, and post-transcriptional gene expression [67,68]. Local inflammation seen in pancreatic cancer (PC) subtypes, such as pancreatic ductal adenocarcinoma (PDAC) is implicated in the process of metastasis [69,70]. Recent findings highlight the lncRNA XLOC_000647 and its impact on inflammasome signalling and EMT in the pathogenesis of PC. XLOC_000647 was found to be significantly downregulated in PC tissues and cell lines. Its expression also inversely correlated with tumour stage, lymph node metastasis, and overall survival [71]. Overexpression of XLOC_000647 suppressed proliferation, invasion, and EMT-related changes in vitro, while attenuating tumour growth in vivo. Mechanistically, luciferase reporter assays in human embryonic kidney cells (293T) demonstrated that XLOC_000647 repressed NLRP3 by reducing promoter activity. Knockdown of NLRP3 inhibited proliferation, invasion, and mesenchymal marker expression such as vimentin, while restoring epithelial traits such as E-cadherin expression. Conversely, ectopic expression of NLRP3 reversed the inhibitory effects of XLOC_000647 on EMT and invasion. These findings affirm NLRP3′s functional role as a downstream effector and novel lncRNA-based epigenetic circuit modulating inflammasome activity in PC [71]. Moreover, lncRNA-inflammasome interactions were identified as a potential target for the modulation of PC dissemination.

#### 2.2.3. Complementary Findings in Colorectal Cancer

Complementing Marandi’s findings in CRC, discussed earlier in the context of intercellular regulation, it has also been demonstrated that the inflammasome sensor AIM2 plays a tumour-suppressive role in human CRC cells by inhibiting EMT via both Akt-dependent and inflammasome-mediated mechanisms [72]. Overexpression of AIM2 led to increased E-cadherin and reduced vimentin and SNAIL expression, correlating with decreased migratory and invasive capacity. Conversely, AIM2 silencing promoted mesenchymal marker expression and Akt phosphorylation. The EMT marker shifts were reversed by inhibition of Akt or caspase-1, highlighting a dual axis through which AIM2 inhibits EMT [72]. Overall, while clinical data associates inflammasome activation with EMT progression, experimental findings indicate that relieving inflammasome inhibition can suppress EMT. This dichotomy highlights the need for a more refined understanding of the cellular context and tissue-specific inflammatory dynamics driving EMT in cancer.

## 3. Therapeutic Targeting of the Inflammasome–EMT Axis

### 3.1. Lung Cancer

Therapeutic targeting of the inflammasome–EMT axis in cancer reveals a convergent theme. Previous work has shown that the inhibition of NLRP3–IL-1β and downstream mitogen-activated protein kinase (MAPK)/NF-κB/TGF-β networks can attenuate EMT-driven invasion and metastasis. However, several factors in tumour biology limit the translational applicability of these approaches, namely: tumour type, stage, and a more complex cellular niche compared to that observed in non-malignant fibrotic disorders. Recent evidence has explored the potential of inhibiting inflammasome signalling to attenuate EMT-driven metastatic progression in non-small-cell lung cancer (NSCLC). One study [73] investigated the anti-inflammatory and anti-metastatic properties of morin, a polyphenolic flavonoid, in LPS and ATP-stimulated A549 and H1299 NSCLC cell lines. Morin significantly suppressed the expression and secretion of pro-inflammatory cytokines IL-1β, IL-18, and IL-6 in a dose-dependent manner. In addition, several key inflammasome components including NLRP3, ASC, and cleaved caspase-1 were downregulated in morin-treated conditions. These molecular changes were associated with a significant reduction in EMT, evidenced by reduced expression of mesenchymal markers and proteolytic proteins including MMP-2, MMP-9, urokinase-type plasminogen activator (u-PA), urokinase-type plasminogen activator receptor (uPAR), and membrane-type 1 MMP. Functional assays demonstrated that morin significantly inhibited cancer cell migration and invasion, likely mediated by the parallel shut-down of the MAPK pathway [73]. As such, NLRP3/MAPK axis is likely a critical player in inflammation-driven EMT in NSCLC. This also demonstrates that morin may be a promising therapeutic candidate capable of disrupting this axis to limit metastatic potential, although this needs to be validated using in vivo studies.

### 3.2. Pulmonary Fibrosis

Recent studies have demonstrated the potential of targeting the inflammasome–EMT axis in various benign disorders, primarily those involving pathological visceral fibrosis. Pulmonary fibrosis (PF) is a progressive interstitial lung disease characterised by excessive extracellular matrix (ECM) deposition and subsequent architectural distortion, reduced lung compliance, and inefficient gas exchange [74]. PF encompasses a heterogeneous group of disorders, with idiopathic pulmonary fibrosis (IPF) being the most prevalent subtype [75]. Patients with IPF are believed to have an increased risk of lung cancer [76], and concomitant disease is associated with poorer clinical outcomes [77]. Aetiologically, PF can also occur secondary to known insults, including autoimmune diseases such as rheumatoid arthritis and systemic sclerosis, environmental and occupational exposures including silica and asbestos, and medications like amiodarone and bleomycin [78,79,80,81]. Key signalling pathways implicated in the pathogenesis of PF include TGF-β, wingless-related integration site (Wnt)/β-catenin, and the PI3K/Akt/mammalian target of rapamycin (mTOR) axis. These networks collectively promote fibrogenesis through the modulation of cellular senescence, EMT, and apoptosis resistance [82,83,84].

Experimental and clinical evidence supports the role of EMT in PF. TGF-β1 treatment in vitro robustly induces EMT in alveolar epithelial cells [84]. Interestingly, it has been revealed that approximately one-third of fibroblast-specific protein FSP1-positive fibroblasts in bleomycin-induced fibrotic lungs are of epithelial origin [85]. Inflammasome-associated cytokines have demonstrated their role in the pathogenesis of bleomycin-induced PF. For instance, the inhibition of IL-18 using IL-18BP prevented the induction of EMT, shown by a reduction in alpha-smooth muscle actin (α-SMA) and an increase in E-cadherin [86].

One study [87] on silica-induced EMT in human bronchial epithelial cells demonstrated that the inhibition of the NLRP3 inflammasome pathway through MCC950 (selective inhibitor), Z-YVAD-FMK (caspase-1 inhibitor), and shRNA knockdown could suppress EMT. This was mechanistically linked to blockade of the transforming growth factor-β-activated kinase 1 (TAK1)-MAPK-SNAIL and NF-κB signalling pathways in the case of MCC950. The use of pirfenidone, an antifibrotic agent approved for IPF, also mitigated silica-induced EMT by inhibiting NLRP3 activation [87].

Berberine is a bioactive alkaloid that has been shown to modulate various biological pathways including PI3K/AKT, mTOR, and Wnt/β-catenin [88,89,90,91]. Demethyleneberberine (DMB) is the primary metabolite of berberine and exhibits higher bioavailability and mitochondrial antioxidant properties [92,93]. In bleomycin-induced PF models, DMB suppressed the activation of the NLRP3 inflammasome, attenuated IL-1β and IL-18 secretion, and reversed mesenchymal marker expression. Importantly, overexpression of NLRP3 partially diminished the therapeutic effects of DMB, suggesting a causal link between inflammasome suppression and EMT inhibition. DMB also inhibited the expression of caspase-11 and gasdermin D, leading the authors to conclude that DMB acts on both the non-canonical and canonical pathways of NLRP3 inflammasome activation [94].

Another agent which has shown efficacy in bleomycin-induced PF is Iguratimod. Iguratimod is a disease-modifying agent approved for the treatment of rheumatoid arthritis in China and Japan [95]. Its exact mechanism of action is not fully understood, but it has been shown to reduce the production or activity of various pro-inflammatory cytokines such as IL-1β and IL-6 [96]. In one study, Iguratimod treatment reduced hydroxyproline content and fibrosis scores in a bleomycin-induced PF mouse model [97]. Iguratimod was shown to inhibit NLRP3, reverse EMT, and suppress the production of ROS [97]. While this study did not specifically establish causation between NLRP3 inhibition and EMT reversal, this link seems highly plausible given the evidence provided by other studies. For instance, a similar effect is seen with scutellarin, a flavonoid derivative extracted from the Erigeron breviscapus plant. In both in vivo and in vitro models of bleomycin-induced PF, scutellarin treatment dose-dependently suppressed NF-κB activation and canonical NLRP3 inflammasome components, reducing downstream IL-1β and IL-18 production [98]. This was accompanied with EMT marker reversal and restoration of E-cadherin expression, alongside reduced collagen I and α-SMA expression and deposition [98].

From a cell therapy perspective, exosomes may provide a promising avenue in bleomycin-induced PF. Exosomes are nano-sized extracellular vesicles released by a variety of cells into the extracellular space, carrying diverse cargo consisting of proteins, lipids, and nucleic acids [99,100]. Thus, exosomes are central players in cell–cell communication and are capable of influencing various biological processes [101]. In a recent study [102], mesenchymal stem cell-derived exosomes (MSC-exos) have shown anti-fibrotic activity. In both in vivo and in vitro models, MSC-exos suppressed the activation of nucleotide-binding oligomerisation domain-containing protein 1 (NOD1)/NF-κB/NLRP3 signalling. In addition, they reduced expression of IL-1β and IL-18 and reversed the expression of mesenchymal markers. Overexpression of NLRP3 significantly attenuated the therapeutic effects of MSC-exos on EMT and ECM deposition [102]. These findings highlight the potential of MSC-exos as a cell-free therapeutic approach targeting NLRP3-driven inflammasome–EMT signalling in PF.

### 3.3. Peritoneal Fibrosis

Peritoneal fibrosis is a major complication and leading cause of discontinuation of peritoneal dialysis in end-stage renal disease [103,104]. The peritoneal mesothelium is a monolayer of specialised epithelial-like cells that line the peritoneal cavity [105]. In peritoneal dialysis, repeated exposure to bioincompatible solutions and inflammatory insults leads to the denudation of this monolayer [106]. EMT has been shown to play a role in this fibrotic condition [107,108]. Some lines of evidence suggest that EMT in peritoneal mesothelial cells is critically mediated by the NLRP3 inflammasome. Stimulation with TGF-β1 triggered EMT in peritoneal cells, as demonstrated by the loss of epithelial markers and acquisition of mesenchymal traits [109]. TGF-β1 stimulation led to increased expression and activation of NLRP3, ASC, and cleaved caspase-1, resulting in elevated IL-1β and IL-18 secretion. Blocking NLRP3, ASC, or neutralising IL-1β/IL-18 significantly reduced EMT-related alterations, indicating that the activation of the inflammasome and its related cytokines drive this phenotypic transition. Mechanistically, TGF-β1 induced both membrane-bound NOX1 activation and mitochondrial NOX4 upregulation, promoting oxidative stress that was believed to act as a proximal signal for inflammasome activation. Crucially, treatment with paricalcitol, a vitamin D receptor agonist, suppressed NOX expression, NLRP3 expression, and EMT [109]. To our knowledge this work is the first to establish an inflammasome–EMT axis in peritoneal cells, and also suggest a therapeutic avenue for the targeting of this pathway.

### 3.4. Adenomyosis and Endometriosis

Endometriosis is a chronic inflammatory disorder characterised by the growth of endometrial-like tissue outside the uterine cavity [110]. Endometriosis may be associated with an increased risk of mainly ovarian cancer [111], but also various other malignancies such as endometrial and breast cancer [112]. Adenomyosis is a related condition defined by the presence of endometrial glands and stroma within the myometrium, resulting in uterine enlargement, heavy menstrual bleeding, and dysmenorrhea [113]. EMT has emerged as a key driver in the development and progression of adenomyosis. Mounting evidence indicates that innate inflammatory signalling, including the inflammasome complex, is upregulated in adenomyosis and may increase disease progression [114]. EMT is also known to be a key driver of adenomyosis in endometrial cells [115]. 17β-oestradiol or E2 exposure has been demonstrated to cause endometrial glandular cells to adopt a fibroblast-like morphology, downregulate epithelial markers, upregulate mesenchymal markers and gain migratory and invasive capabilities [114,116]. Increased expression of oestrogen receptor beta (ER-β) has been demonstrated to activate the inflammasome, promoting cell invasion and proliferation [117].

It has previously demonstrated that blockage of the NLRP3 inflammasome by MCC950, a diary sulfonylurea-containing compound, attenuated the invasive capacity of endometrial cells. Additionally, in a murine model of adenomyosis, MCC950 reduced the severity of adenomyosis [118]. Other studies have confirmed these effects in similar models, demonstrating that the downregulation of gene associated with retinoid-interferon-induced mortality 19 (GRIM-19), a mitochondrial protein in macrophages, triggers NLRP3 inflammasome activation, which results in macrophage pyroptosis and elevated IL-1β secretion, promoting proliferation and migration of endometrial cells. Neutralising IL-1β reversed these effects, confirming the inflammasome’s role in adenomyosis and endometrial cell migration [119].

Additionally, the NLRP3 inflammasome has been implicated in the process of invasion and migration in endometriosis. In patients with endometriosis, peritoneal fluid washings demonstrate higher levels of caspase 1, IL-1β and gasdermin D (GSDMD) compared with healthy individuals. IL-1β concentrations are also associated with increased disease severity. The treatment of endometrial stromal cells with IL-1 also increased the migratory ability of ESCs, increasing concentrations of MMP-1 and MMP-3 [120]. TRIM24 is a negative regulator of NLRP3/caspase-1/IL-1β-mediated migration. In cells stimulated by the inhibition of TRIM24 with TRIM24-small-interfering RNA, the promoted pyroptosis and cell migration was reversed by CY-09, a specific inhibitor of NLRP3. This further implicates this inflammasome in the process of EMT in endometriosis [121].

### 3.5. Diabetic Nephropathy

Targeting the inflammasome–EMT axis has shown promise in the treatment of diabetic nephropathy (DN) in pre-clinical studies. Using a DN rat model induced by unilateral nephrectomy and intraperitoneal streptozotocin injection, one study demonstrated an upregulation of the NLRP3 inflammasome, its downstream effectors, and the expression of mesenchymal markers [122]. The study further revealed that treatment with Huangkui capsule, a traditional anti-inflammatory phytomedicine extracted from the Abelmoschus Manihot plant, significantly attenuated renal tubular EMT by suppressing NLRP3 inflammasome activation. This inhibition was likely mediated by the upstream blockade of the TLR4/NF-κB signalling pathway [122]. However, although the study suggests that TLR4/NF-κB signalling acts upstream of NLRP3, causal relationships were inferred rather than directly demonstrated. Therefore, we suggest that the use of pathway-specific inhibitors or gene knockdown models in the future would strengthen mechanistic conclusions in DN. In a separate study on DN rat models, the Huangkui capsule was shown to activate the peroxisome proliferator-activated receptor-α/γ and attenuated endoplasmic reticulum stress in rats [123]. Further studies should aim to elucidate the pathways modulated by the Huangkui capsule in DN more clearly, particularly with regard to modulation of the inflammasome–EMT axis.

Another possibility for the targeting of inflammasome–EMT signalling in DN is through berberine. One study laid out in vivo and in vitro evidence that berberine ameliorates diabetic nephropathy by inhibiting renal tubular EMT through suppression of the NLRP3 inflammasome [124]. In a streptozotocin-induced diabetic rat model, berberine treatment significantly recued pathological findings on kidney samples as shown by decreased markers of interstitial fibrosis, and the reversal EMT-associated changes. In vitro experiments in high-glucose-stimulated HK-2 cells confirmed that berberine downregulated both protein and mRNA levels of NLRP3, ASC, caspase-1, and IL-1β in a dose-dependent manner [124]. These findings link NLRP3 inflammasome activation and EMT progression in DN. They also support berberine’s therapeutic potential as an inhibitor of inflammasome-mediated EMT and fibrosis.

**Figure 3:**
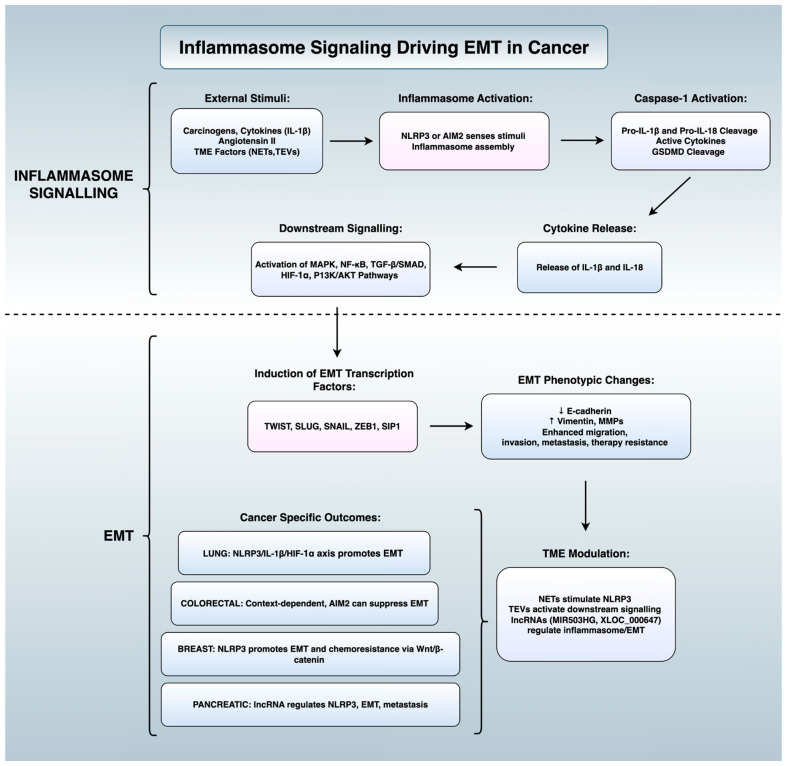
Schematic representation of inflammasome signalling driving EMT in cancer. External stimuli activate inflammasomes, leading to downstream signalling, cytokine release, and induction of EMT transcription factors, which promote phenotypic changes, tumour microenvironment modulation, and site-specific outcomes. Figure created using BioRender (version 2025, BioRender.com, Toronto, ON, Canada) and Microsoft PowerPoint (Microsoft Corporation, version 365, Redmond, WA, USA).

**Figure 4:**
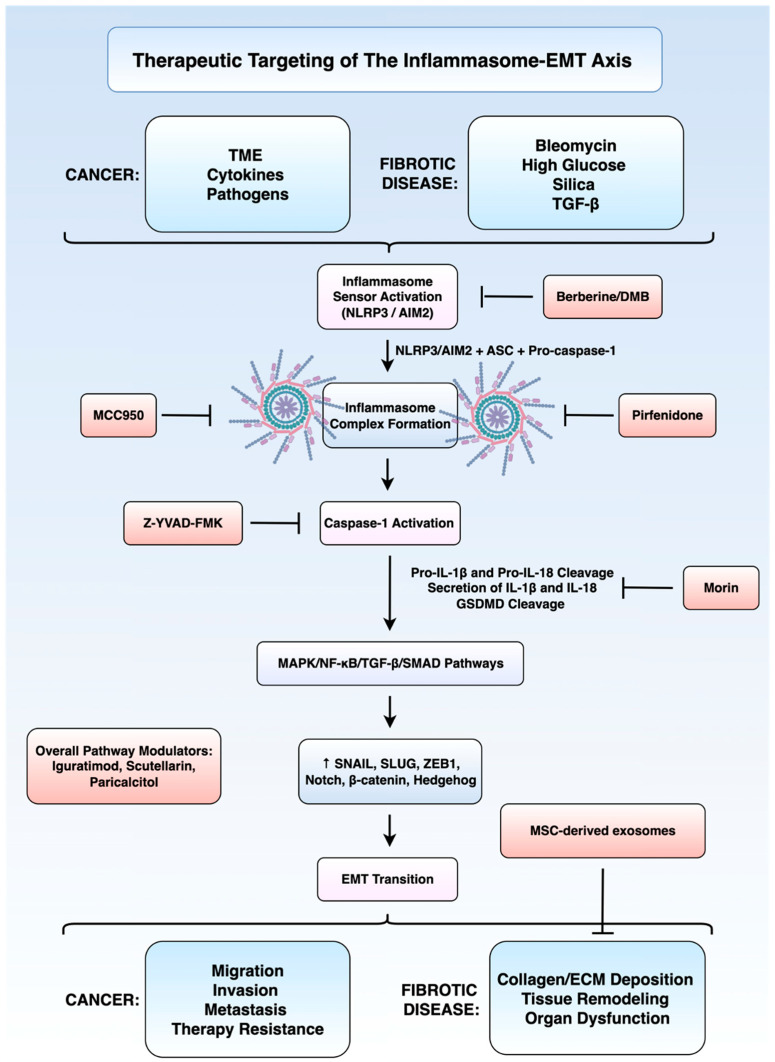
Therapeutic targeting of the inflammasome–EMT axis in cancer and fibrotic disease. Figure created using BioRender (version 2025, BioRender.com, Toronto, ON, Canada) and Microsoft PowerPoint (Microsoft Corporation, version 365, Redmond, WA, USA).

In summary, across cancer and fibrotic diseases, inflammasome inhibition can curb EMT by disrupting NLRP3-dependent IL1β signalling and its engagement of MAPK, NF-κB, and TGF-β/Smad pathways in order to achieve particular treatment goals. In cancer, the therapeutic benefit is to halt EMT driven invasion, metastasis, and associated therapy resistance, with a focus on tumour type, microenvironmental modulators (e.g., NETs, TEVs), and sensor specificity (NLRP3 versus AIM2 oligomerisation) while also preserving normal host immune defences. In non-malignant diseases, the priority is to prevent or reverse EMT associated fibrosis and organ remodelling, often with broader safety margins and potential for combination with antifibrotic agents or cell-based therapies. Figure 3 summarises the mechanisms driving the interplay between the inflammasome and the EMT axis, while Figure 4 outlines the therapeutic agents being explored for their potential in modulating this pathway in cancer and fibrotic disorders.

## 4. Beyond EMT: The Inflammasome as a Driver of Endothelial-Mesenchymal Transition

The endothelial-mesenchymal transition (EndoMT) has emerged as another NLRP3-driven source of mesenchymal cells in fibrosis. One study [125] investigated this phenomenon using a bleomycin-induced lung injury model in C57BL/6 mice and complementary in vitro experiments in human pulmonary microvascular endothelial cells (HPMECs). Bleomycin markedly upregulated NLRP3, cleaved caspase-1, and IL-1β. These changes were joined by loss of endothelial markers VE-cadherin and CD31 and the acquisition of mesenchymal properties. Moreover, pharmacological inhibition of caspase-1 with Ac-YVAD-cmk (YVAD) decreased NLRP3 and mesenchymal marker expression, restored the expression of endothelial markers, and improved pathological indices of lung injury and fibrosis on histological specimens. Mechanistically, the study identified focal adhesion kinase (FAK) phosphorylation as a key downstream effector in this process. Bleomycin increased the p-FAK/FAK ratio in vivo and in vitro, whereas YVAD treatment reversed these effects [125]. These findings suggest a pathogenic sequence in which NLRP3 inflammasome activation promotes caspase-1-dependent FAK activation, driving EndoMT and contributing to fibrotic remodelling. Similar endothelial involvement was observed in a study on mechanical stretch-induced fibrosis, where ventilator overdistension triggered NLRP3 activation, EndoMT marker shifts, and collagen accumulation. These effects were largely prevented in NLRP3-deficient mice or following NLRP3 knockdown in stretched endothelial cells [126]. These results suggest a NLRP3-EndoMT pathway in ventilator-associated fibrosis.

## 5. Inflammasome–EMT Signalling as a Danger-Plasticity Axis

It is widely acknowledged that chronic low-grade inflammation is a major enabling characteristic of cancer [127,128]. Tumour-promoting inflammation supports the malignant phenotype by enhancing genomic instability, cell proliferation, angiogenesis, invasion, and immune evasion through the sustained release of secreted factors and ROS [129,130]. We therefore propose that the discussed interplay between EMT and inflammasome activation can be conceptualised as a ‘danger-plasticity’ axis of evolutionarily conserved cellular programmes. That is, in cases of severe injury, cells respond by activating inflammasomes, which in turn initiate the pyroptosis pathway. This response can be protective in acute injury, and may function to clear damaged or potentially malignant cells. In contrast, under sublethal cellular stress and a survivable level of inflammation, activation of the EMT programme may influence cells towards a mesenchymal phenotype capable of tissue remodelling and repair. Within this adaptive shift, loss of E-cadherin occurs, which would typically push epithelial cells into a form of programmed cell death known as anoikis [131,132]. However, in EMT, loss of E-cadherin is accompanied by the upregulation of various anti-apoptotic mediators like B-cell lymphoma-extra large (BCL-xL) and survivin [133,134]. These transcriptional programmes enhance the capacity of these mesenchymal-like cells to persist and function despite sustained inflammatory or metabolic stress. This evolutionary trade-off may serve an important physiological role in wound healing and tissue remodelling, but may similarly be advantageous to cancer cells, allowing them to acquire invasive and metastatic capabilities. Harold F. Dvorak famously likened tumours to ‘wounds that do not heal’ [135], a description that resonates strongly with the persistent, dysregulated interplay between inflammation and phenotypic adaptation seen in cancer. As such, the inflammasome could operate as a central decision-making node on this axis, by integrating the intensity and persistence of stress cues in order to direct cells towards either elimination or adaptive reprogramming.

## 6. The Future of Translational Research in the Inflammasome–EMT Landscape

Proteins involved in the inflammasome and EMT pathways have been explored as potential prognostic biomarkers for various malignancies. For example, in gastric cancer, the stratification of different molecular phenotypes including EMT status, corresponds to pathological tumour characteristics such as histological subtype, advanced stage, metastatic nodal foci and peritoneal spread [136]. In a separate study, NLRP1/NLRP3 expression was linked to poor overall survival, advanced stage, the development of lymph node metastases, and the infiltration of immune cells in the tumour microenvironment [137]. The latter study demonstrated that NLRP3 is able to bind to the promoter region of the cyclin D1 gene, enhancing its transcription. Signalling pathways involving cyclin D1 have previously been implicated in the development of EMT in gastric cancer and other cancers [138,139]. Thus, despite the separate study of these two molecular pathways in prognostication, their biological links suggest that their combined use could be effective in predicting patient outcomes and therapy responses, which warrants further investigation. In terms of therapeutic potential, through the inhibition cyclooxygenase-mediated prostaglandin E2 production, aspirin was able to downregulate inflammasome activity [140]. In a separate study, it has been shown that the effects of aspirin on inhibiting platelet function could be harnessed to prevent EMT-promoting platelet-circulating tumour cell (CTC) interactions [141]. In terms of immunotherapy, past data has shown that interrupting the actions of cluster of differentiation 73 (CD73), macrophage colony stimulating factor 1 (CSF1) or osteopontin (SPP1) on partially mesenchymal cells, a cellular intermediates of the EMT process, could reverse the immunosuppressive tumour niche [136,142,143]. These partially mesenchymal cell populations targeted have previously been identified in patients bearing pancreatic cancer [136]. The interactions of markers such as CD73 and inflammasome activity have been shown in previous papers studying neuroglial cells; however, this mechanism has not been supported in other organs or in malignant cells [144]. SPP1 has recently been identified as a critical regulator of the mesenchymal identity of pancreatic carcinoma progenitor cells [143]. While its connections with inflammasome activity have not been previously delineated in malignant tumours, in the central nervous system, the priming step of NLRP3 activation may lead to NF-κB-mediated transcription of SPP1 [145]. Given that the separate components of the inflammasome–EMT axis can be modulated by the same therapies, it is reasonable to assume that further explorations could potentially establish connections between them, and to maximise anticancer therapeutic effects.

In order to strengthen the translational research front in the inflammasome-EMT area, novel laboratory techniques studying these processes could be employed. Traditional methods used to study EMT include the formation of cell-line derived xenografts or co-culturing malignant cell lines with EMT-inducing or restricting secreted factors [146]. Novel techniques such as 3D spheroidal tumour models could reveal differential gene expression patterns in various tumour cell lines as well as different inflammasome dynamics within those cells [146,147]. This process could be further refined with patient-derived organoids which allow for the consideration of the diverse non-malignant cellular milieu of the tumour microenvironment [146,148]. Inflammasome related genes have already been studied in organoids from patients with benign inflammatory disorders such as ulcerative colitis, opening up a prospective investigation in the context of malignancy [149]. Overall, despite the successes of traditional laboratory methods, the employment of such novel modelling techniques could further refine our understanding of the complex inflammasome–EMT axis in multiple primary cancer sites, and the microenvironments of various metastatic organs.

## 7. Conclusions

The inflammasome–EMT axis is a critical regulator of cancer progression. NLRP3 activity is central to this relationship in multiple tumour types, due to its role in coupling inflammasome activation to EMT via IL-1β–dependent signalling that engages NF-κB, TGF-β/Smad, and Wnt/β-catenin pathways. The AIM2 inflammasome, on the other hand, can either promote or attenuate the EMT process depending on the tumour type. This phenomenon is well-characterised in colorectal cancer where inflammasome activity sometimes promotes the EMT, while on other occasions can restrict EMT. These tissue-specific changes are probably explained by differences in the tumour niche, as well as molecular regulators such as RNAs, epigenetic changes and post-transcriptional modifications which control inflammasome components. The tumour microenvironment also contributes to this bidirectional inflammasome–EMT relationship, with inflammatory mediators and NETs amplifying inflammasome signalling in tumour and stromal cells, and in turn, promoting dissemination. From a clinical point of view, the effects of EMT driven by inflammasome activity have been linked to therapeutic resistance. For instance, in TNBC, NLRP3—IL-1β—Wnt signalling promotes both EMT and chemoresistance. The translation of these findings into a safe and effective therapeutic depends on a tumour-specific approach which suppresses EMT, while upkeeping the role of the in-flammasome in host defence and tissue repair.

## Data Availability

Not applicable.

## Abbreviations

The following abbreviations are used in this manuscript:

AIM2	Absent in Melanoma 2
α-SMA	Alpha-Smooth Muscle Actin
ASC	Apoptosis-Associated Speck-Like Protein Containing a Caspase Recruitment Domain
BCL-xL	B-Cell Lymphoma-Extra Large
CARD	Caspase Recruitment Domain
CD31	Cluster of Differentiation 31
CD73	Cluster of Differentiation 73
CSC	Cancer Stem Cell
CSF1	Colony Stimulating Factor 1
CTC	Circulating Tumour Cell
DAMPs	Damage-Associated Molecular Patterns
DMB	Demethyleneberberine
DN	Diabetic Nephropathy
ECM	Extracellular Matrix
EMT	Epithelial-Mesenchymal Transition
EndoMT	Endothelial-Mesenchymal Transition
FAK	Focal Adhesion Kinase
FSP-1	Fibroblast-Specific Protein 1
GSDMD	Gasdermin D
HIF-1α	Hypoxia-Inducible Factor 1 Alpha
HPMECs	Human Pulmonary Microvascular Endothelial Cells
IKKα	Inhibitor of Nuclear Factor Kappa-B Kinase Subunit Alpha
IKKβ	Inhibitor of Nuclear Factor Kappa-B Kinase Subunit Beta
IκBα	Inhibitor of Kappa B Alpha
IL-1β	Interleukin-1 Beta
IL-18	Interleukin-18
IL-18BP	Interleukin-18 Binding Protein
IPF	Idiopathic Pulmonary Fibrosis
lncRNA	Long Non-Coding RNA
LPS	Lipopolysaccharide
MAPK	Mitogen-Activated Protein Kinase
MET	Mesenchymal-Epithelial Transition
MMPs	Matrix Metalloproteinases
MSC	Mesenchymal Stem Cell
MSC-exos	Mesenchymal Stem Cell–Derived Exosomes
mTOR	Mammalian Target of Rapamycin
MyD88	Myeloid Differentiation Primary Response 88
NEMO	NF-κB Essential Modulator
NEK7	Never in Mitosis Gene A–Related Kinase 7
NETs	Neutrophil Extracellular Traps
NF-κB	Nuclear Factor Kappa-Light-Chain-Enhancer of Activated B Cells
N-GSDMD	N-Terminal Fragment of Gasdermin D
NLRC4	NOD-Like Receptor Family CARD Domain–Containing 4
NLRP1	NOD-Like Receptor Family Pyrin Domain–Containing 1
NLRP3	NOD-Like Receptor Family Pyrin Domain–Containing 3
NOD1	Nucleotide-Binding Oligomerisation Domain-Containing Protein 1
NOX4	Nicotinamide Adenine Dinucleotide Phosphate Oxidase 4
NSCLC	Non-Small-Cell Lung Cancer
PAMPs	Pathogen-Associated Molecular Patterns
PF	Pulmonary Fibrosis
PI3K/AKT	Phosphoinositide 3-Kinase/Protein Kinase B
PRR	Pattern Recognition Receptor
PYD	Pyrin Domain
ROS	Reactive Oxygen Species
SIP1	Smad-Interacting Protein 1
SNAIL	Snail Family Transcriptional Repressor 1
SLUG	Snail Family Transcriptional Repressor 2
SMAD	Homologs of Mothers Against Decapentaplegic
SPP1	Osteopontin
TAK1	Transforming Growth Factor-β–Activated Kinase 1
TGF-β	Transforming Growth Factor Beta
TLR4	Toll-Like Receptor 4
TME	Tumour Microenvironment
TNBC	Triple-Negative Breast Cancer
TNF-α	Tumour Necrosis Factor Alpha
TNFR	Tumour Necrosis Factor Receptor
TRAF	TNF Receptor-Associated Factor
TRIF	Toll/Interleukin-1 Receptor Domain-Containing Adaptor-Inducing Interferon-β
TWIST	Twist-Related Protein 1
uPA	Urokinase-Type Plasminogen Activator
uPAR	Urokinase-Type Plasminogen Activator Receptor
Wnt	Wingless-Related Integration Site
YVAD	Ac-YVAD-cmk
ZEB1	Zinc Finger E-Box Binding Homeobox 1
ZO1	Zonula Occludens-1
